# Unexpected prey of juvenile spotted scat (*Scatophagus argus*) near a wharf: The prevalence of fouling organisms in stomach contents

**DOI:** 10.1002/ece3.4380

**Published:** 2018-07-30

**Authors:** Xianzhi Lin, Simin Hu, Sheng Liu, Hui Huang

**Affiliations:** ^1^ Key Laboratory of Tropical Marine Bio‐resources and Ecology Guangdong Provincial Key Laboratory of Applied Marine Biology South China Sea Institute of Oceanology Chinese Academy of Sciences Guangzhou China; ^2^ University of Chinese Academy of Sciences Beijing China; ^3^ Tropical Marine Biological Research Station in Hainan Chinese Academy of Sciences Sanya China

**Keywords:** 18S rDNA, dietary analysis, high‐throughput sequencing, juvenile fish

## Abstract

A knowledge of fish diets can contribute to revealing the trophic role and ecological function of species in aquatic ecosystems. At present, however, there are no efficient or comprehensive methods for analyzing fish diets. In this study, we investigated the diets of juvenile *Scatophagus argus* collected near a wharf in Daya Bay, China, by dissection and high‐throughput sequencing (HTS) using the 18S rDNA V4 region. Microscopy disclosed large amounts of bryozoans and unrecognizable detritus. In contrast, HTS analysis indicated that the fish diets were considerably more diverse than visual inspection suggested. After eliminating fish sequences, approximately 17,000 sequences from taxa in nine phyla (Ciliophora, Bryozoa, Annelida, Bacillariophyta, Chlorophyta, Arthropoda, Dinoflagellata, Tunicata, and Phaeophyta) were identified from the analysis of stomach contents. Twenty‐one food categories were identified, most of which (95.2%) were benthic fouling organisms that could easily be collected around wharfs. These consisted of bryozoans (31.9%), ciliates (45.7%), polychaetes (14.6%), and green algae (3.0%). Therefore, to adapt to anthropogenic habitat modification, the fish had probably shifted from planktonic to benthic feeding. The prevalence of fouling organisms in the stomachs of juvenile *S. argus* indicates that the fish have responded to habitat changes by widening their food spectrum. This adaptation may have increased their chances of survival. The fouling organisms that inhabit highly perturbed coastal ecosystems could represent a food source for animals at higher trophic levels. Our results accordingly suggest that human activity might significantly influence fish feeding behavior and material transfer along the food chain.

## 1 INTRODUCTION

Fish are important consumers in aquatic ecosystems, in which they contribute to the maintenance of homeostasis by driving top‐down forces and trophic cascades (Leray, Meyer, & Mills, 2015; Weber & Traunspurger, 2015). Information concerning fish dietary supplementation in the field is vital for pisciculture (Robert, Levesque, Gagne, & Fortier, 2011). Fish have diverse diets and occupy multiple trophic levels. They display various feeding patterns, such as planktonic and benthic (Gupta, 2016; Riemann et al., 2010), and can also adjust their feeding behaviors according to food availability in the natural environment (Prudente, Carneiro‐Marinho, Valente, & Montag, 2016). One such example is *Serrasalmus gouldingi* (Fink & Machado‐Allison 1992), which is the most abundant fish species in the Anapu River of the Amazon Basin (Prudente et al., 2016). The juveniles of this species have narrow food niches during droughts and early during periods when the water level rises, and at these times, they mainly consume other fish species. However, when the water recedes, their diet becomes more varied and they consume a larger proportion of indigenous insects and other arthropods (Prudente et al., 2016). Fish with such wide trophic niches and flexible feeding strategies adapt better to environmentally driven fluctuations in food resources (Moreno‐Valcarcel, Oliva‐Paterna, Bevilacqua, Terlizzi, & Fernandez‐Delgado, 2016; Prudente et al., 2016). The diets of marine fish may reflect both environmental conditions and their survival status, and therefore, in situ fish diet studies may help us to understand their survival strategies. This knowledge is particularly important for juvenile fish because they are critical indices of successful resource management and artificial breeding in fisheries and aquaculture (Robert et al., 2011).

Coastal ecosystems are essential to marine ecological services and have also been a focal point of the conflict between marine exploitation and environmental protection. Anthropogenic disturbances have replaced the natural coastal water habitats with mosaic landscapes, and this transformation has influenced the availability of food resources and material transfer along the food chain in coastal ecosystems (Anderson & Cabana, 2009; Huang, Zhang, & Jiang, 2015). Consequently, native organisms may be forced to change their survival strategies, including their feeding habits (Quéméré et al., 2013). In this regard, Anderson and Cabana (2009) reported that the slopes of the δ^15^N‐size class relationships in the invertebrate community changed from −1 to +2.8 as a consequence of industrial activity at 23 river sites. Nevertheless, it remains difficult to assess the impact of human activity on the trophic relationships in these ecosystems because accurate dietary information is often lacking. The ecological processes involved in these food chain shifts are also uncertain because there is typically limited information available regarding trophic dynamics. For these reasons, it is necessary to gain an understanding of the changes in food sources and feeding behaviors exhibited by consumers like fish in response to environmental disturbance (Leray et al., 2015). Although several studies have previously focused on the feeding behavior of larval or juvenile fish, the information derived from this research remains incomplete because of limitations associated with the available sampling and analytical methods (Paradis, Sirois, Castonguay, & Plourde, 2012; Robert et al., 2011).

Conventional fish diet analysis is based on the morphological identification of preys in the gut contents or feces. Nevertheless, when using this approach, a specialized knowledge of taxonomy is required to enable researchers to identify prey accurately. The partial digestion of soft‐bodied organisms makes it even more difficult to identify prey correctly (Deagle, Kirkwood, & Jarman, 2009; Schuckel et al., 2013). In addition, the presence of the indigestible remains of hard‐bodied organisms introduces error into dietary assessments (Blankenship & Yayanos, 2005; Weber & Traunspurger, 2014). In contrast, molecular DNA‐based identification methods are sensitive, rapid, and accurate and have been widely used in dietary analysis (King, Read, Traugott, & Symondson, 2008). They are particularly suitable for small predators like larval or juvenile fish, and the prey of which are generally very small and difficult to characterize (Hu et al., 2014; Maloy, Culloty, & Slater, 2013). High‐throughput sequencing (HTS) is a powerful dietary study tool because it provides comprehensive sequence information at relatively low cost (Pompanon et al., 2012). The sequence data generated by HTS significantly expands existing knowledge on the food spectra of different predators because this technique can detect very rare prey species (Deagle et al., 2009).

In this study, HTS was applied in an in situ dietary analysis of juvenile *Scatophagus argus*(Linnaeus 1766) collected from Daya Bay, Guangdong, China, a region that is under severe threat from human activity (Huang et al., 2015). *Scatophagus argus* is a popular aquarium fish worldwide and a commercially important aquatic species in south and Southeast Asia (Gupta, 2016). Previous dietary studies on *S. argus* have mainly been based on traditional methods (like morphological identification on gut remaining and feeding experiment), and the precise composition of the diet of this fish has yet to be clarified. Most studies have reported that *S. argus* is omnivorous but has a preference for phytoplankton (Gandhi, 2002; Sivan & Radhakrishnan, 2011). It has also been stated elsewhere that *S. argus* is omnivorous but displays a flexible survival strategy in its natural environment (Wongchinawit, 2007). The objectives of this study were to identify and characterize the food resources of juvenile *S. argus*, and to assess the changes in these resources in response to long‐term anthropogenic disturbances in Daya Bay. Furthermore, we sought to provide insight into the trophic role of juvenile fish in coastal marine ecosystems. This information could be applied to the artificial breeding of this species.

## 2 MATERIALS AND METHODS

### 2.1 Sample collection

**Figure 1:**
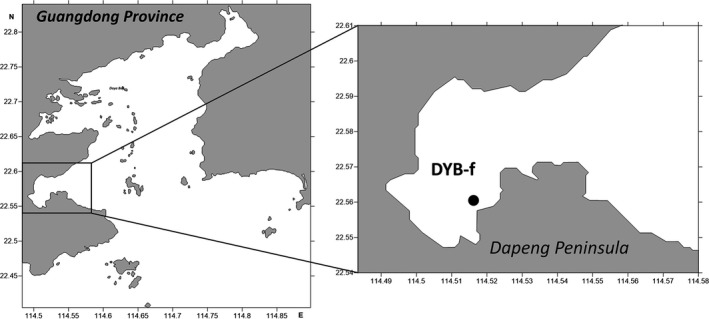
Sampling site (DYB‐f) near a wharf in Daya Bay, Guangdong, China, close to human habitation

Juvenile *S. argus* (standard body length 15.36 ± 2.51 mm) were manually sampled near a wharf in Daya Bay (DYB‐f: 22°33′15.45′′N, 114°31′08.64′′E) on two consecutive nights in September 2015 (Figure 1). The fishes were euthanized by cold shock before preserved in 95% v/v ethanol and stored at 4°C.

### 2.2 Photography of stomach contents and DNA extraction

Approximately 30 juveniles of *S. argus* were dissected, and their stomach contents were carefully collected. These were photographed under a dissecting microscope fitted with a digital camera (Olympus U‐TV0.5XC‐3; Olympus Corp., Shinjuku, Tokyo, Japan) to validate the data obtained from HTS. The stomach contents were rinsed with phosphate‐buffered saline (PBS: 0.01 M, pH range 7.2–7.6) and then macerated with ground glass. The resulting homogenates were resuspended in lysis buffer for 48 hr at 55°C. DNA was extracted from the homogenates following a modified cetyl trimethylammonium bromide (CTAB) protocol (Zhang & Lin, 2002) and eluted in 30 μl 10 mM Tris–HCl (pH 8.0).

### 2.3 Acquisition of the full‐length sequence of *S. argus* 18S rDNA

The genomic DNA from juvenile *S. argus* muscle was extracted, and full‐length 18S rDNA was amplified using universal primers (18S comF1: 5ʹ‐GCTTGTCTCAAAGATTAAGCCATGC‐3ʹ, 18S comR1: 5ʹ‐CACCTACGGAAACCTTGTTACGAC‐3ʹ) (Zhang & Lin, 2002). PCR was run in a 25 μl volume consisting of 2.5 μl (2.5 μM) 10× Taq buffer, 2 μl (2.5 μM) dNTP Mix, 1 μl genomic DNA, 1 μl (5 μM) each of universal forward and reverse primers, 0.125 μl (2.5 U/ μl) ExTaq polymerase (TaKaRa Ex Taq ^®^ Hot Start Version; TaKaRa Bio Inc., Kusatsu, Shiga, Japan), and 17.375 μl dd H_2_O. The PCR conditions were as follows: an initial denaturation step at 94°C for 3 min; five cycles of denaturation at 94°C for 20 s, annealing at 52°C for 30 s, and extension at 72°C for 1 min; 30 cycles of denaturation at 94°C for 20 s, annealing at 56°C for 30 s, and extension at 72°C for 1 min; and a final elongation at 72°C for 7 min. The PCR products were examined by electrophoresis in 1% agarose gel, purified, and sequenced.

### 2.4 PCR amplification and sequencing

To ensure data accuracy and reliability, the PCR products were assessed before being sequenced. In some studies, PCR has been used to amplify prey DNA rather than that of the predator (Hu et al., 2014; Maloy et al., 2013); however, this could result in the DNA of certain potential prey being overlooked. Therefore, in this study, whole‐DNA extracts were amplified using the universal TAReuk454FWD1‐TAReukREV3 primer pair (TAReuk454FWD1: 5ʹ‐CCAGCASCYGCGGTAATTCC‐3ʹ; TAReukREV3: 5ʹ‐CTTTCGTTCTTGATYRA‐3ʹ), which targets the V4 region (~380 bp) of the eukaryotic 18S rDNA (Stoeck et al., 2010). 18S rDNA was selected because it has been widely used for the species‐level identification of eukaryotic organisms (Hu et al., 2014; O'Rorke, Lavery, & Jeffs, 2012). PCR was carried out in a 20‐μl reaction volume composed of 4 μl 5× FastPfu Buffer, 2 μl 2.5 mM dNTPs, 0.8 μl each of 5 μM universal forward and reverse primers, 0.4‐μl FastPfu Polymerase, and 10‐ng genomic DNA. The PCR conditions were as follows: an initial denaturation step at 95°C for 5 min; 27 cycles of denaturation at 95°C for 30 s, annealing at 55°C for 30 s, and extension at 72°C for 45 s; and a final elongation at 72°C for 10 min. The PCR products were prepared for sequencing at 10°C, examined by electrophoresis with 2% agarose gel, and then sequenced using the Illumina MiSeq/HiSeq platform (Illumina, San Diego, CA, USA).

### 2.5 Bioinformatics processing of raw sequences

The raw data obtained from the Illumina MiSeq/HiSeq platform were subjected to quality control in accordance with the Illumina MiSeq/HiSeq platform workflow (Pompanon et al., 2012). After data splitting, removing primers sequences, and splicing paired‐end reads, the tags were filtered and intercepted. Only high‐quality, long (>300 bp) sequences remained. Finally, effective tags were obtained for further analysis.

### 2.6 Taxonomic assignment

To evaluate prey composition and diversity, the effective tags were clustered into operational taxonomic units (OTUs) with a 97% threshold. Those OTUs with a frequency of <10 were removed. Representative OTU sequences were aligned to GenBank sequences with BLAST (Basic Local Alignment Search Tool). The five top‐scoring BLAST hits were returned. If any of these were uncultured or unannotated sequences, then the next would be selected until a specific sequence series was compiled. A species name was assigned only if a single best hit achieved 100% similarity, and all the others were ≤99%. A genus name was accepted only if the similarities of the five best hits were ≥98%. A family name was retained only if the similarities of all the best hits were ≥95%. Sequences with maximum similarities <95% were labeled “NA (No Account).” These were most likely the products of PCR errors, contamination, or GenBank deficiency. The number of effective tags was then returned for each phylum. To reduce the risk of misidentification at the lower taxonomic levels, a proportion of the different OTUs was returned for each level and those labeled “NA” were removed prior to dietary composition analysis.

## 3 RESULTS

### 3.1 Microscopic identification of bryozoan‐dominated stomach contents

**Figure 2:**
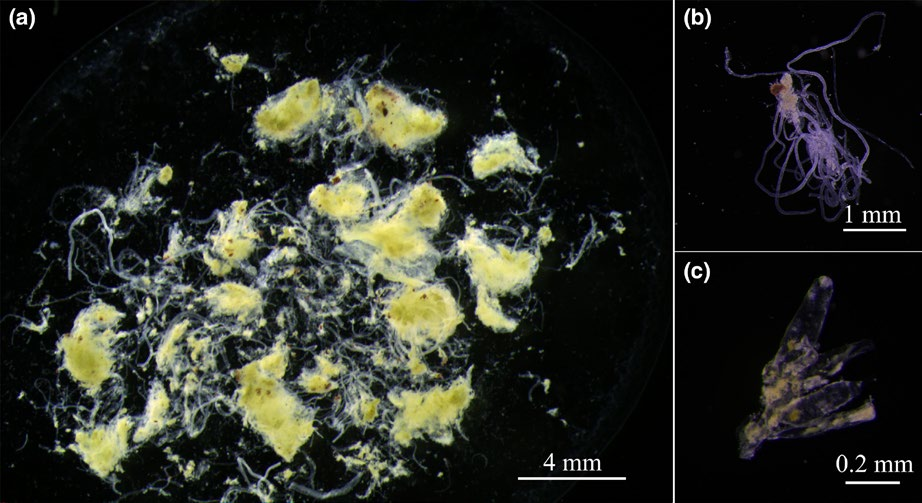
Diet composition of juvenile *Scatophagus argus* based on morphological observations. (a) The stomach contents of juveniles examined under a dissecting microscope, 8× magnification. The contents were difficult to identify accurately without a thorough knowledge of taxonomy. (b) (20× magnification) and (c) (40× magnification). Bryozoan colonies in stomach contents. These were the only recognizable food source detectable by microscopy

Prior to molecular analysis, we conducted microscopic identification of the stomach contents of juvenile *S. argus*. Despite thorough washing, the contents were still difficult to analyze because of the large quantity of flocculent detritus they contained (Figure 2a). For most of the individuals, various forms of bryozoans were the most abundant organisms in the stomach contents (Figure 2b,c).

### 3.2 HTS identified the predominant fouling organisms in *S. argus* juvenile stomachs

**Figure 3:**
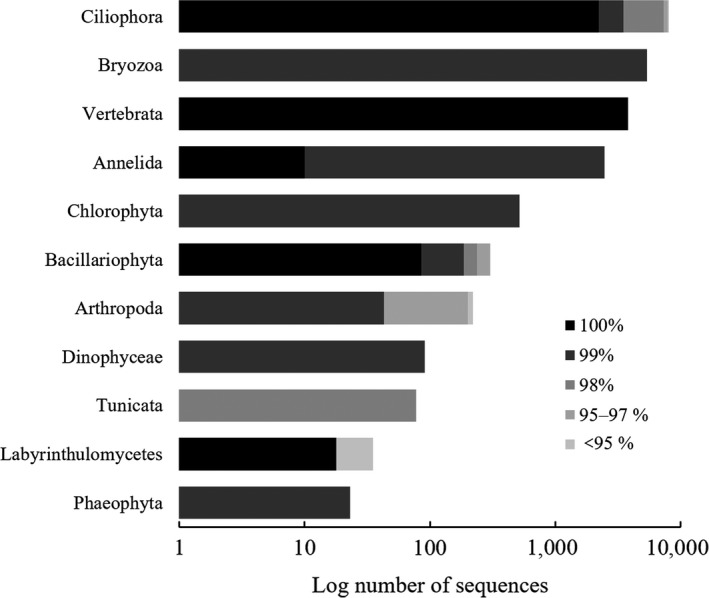
Number and composition of similar sequences assigned to each phylum. High similarity indicated that most food sources could be identified accurately

**Figure 4:**
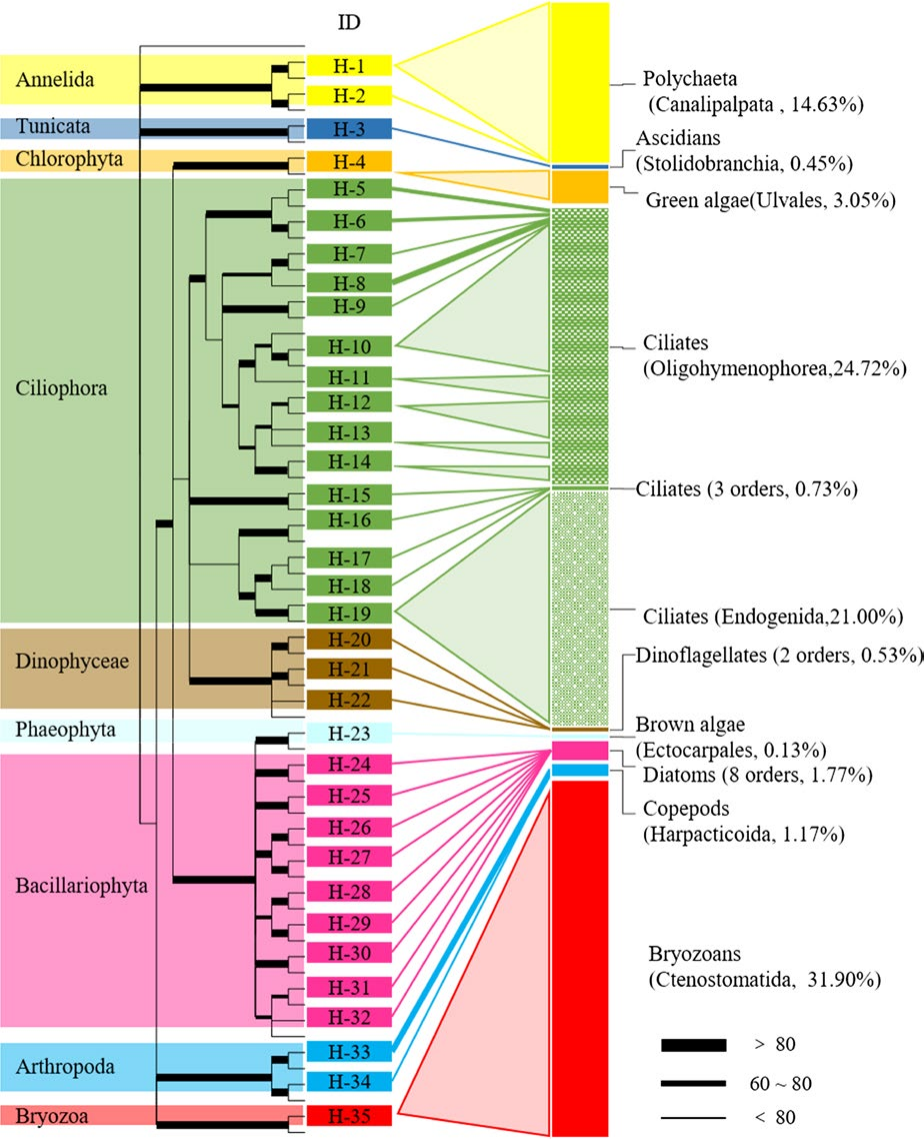
Dietary partitioning and composition of *Scatophagus argus* juveniles. The left part of the figure shows a maximum‐likelihood tree of the 18S rDNA sequence based on high‐throughput sequencing. The 35 operational taxonomic units used in the dietary analysis are shown in the center of the figure. Ciliophora was the most diverse food taxon. The right part of the figure shows the identity of the dietary composition at the order level. Matching colors indicate that the same phyla (Ciliophora, Bryozoa, and Annelida) were the most abundant food sources

A total of 20,974 sequencing reads were returned by the Illumina MiSeq/HiSeq platform. These passed the stringent quality control requirement that neither the multiplex identifier tags (MIDs) nor the primers had any mismatched bases. The BLAST search showed high‐resolution taxonomic assignments (≥98% similarity) for 95.38% of the effective reads (Figure 3). These reads were clustered into 42 OTUs, including seven OTUs identified to the species level, 20 OTUs to the genus level, seven OTUs to the family level, and three OTUs to higher taxonomic levels. Only five OTUs remained unidentified (Table S1). The 42 OTUs spanned 11 different phyla, among which Ciliophora (38.31%), Bryozoa (25.77%), Vertebrata (18.07%), and Annelida (11.83%) were the dominant phyla in the stomach contents of juvenile *S. argus* (Figure 3). The fish DNA sequences were removed during the dietary analysis. Similarly, the unidentified OTUs, labeled “NA,” were removed and assigned to Labyrinthulomycetes, which are unlikely food sources. A total of 35 OTUs remained for downstream analysis (Figure 4).

Strikingly, all the dominant food items in the diet were common fouling organisms. *Bowerbankia*‐like species (99% identity to *Bowerbankia* sp. KM373516.1) were the most abundant group, consisting of 31.90% of the detected diets. Other fouling organisms from genus *Zoothamnium*,* Acineta*,* Branchiomma,* and *Ulva* were also presented in the diet.

## 4 DISCUSSION

The HTS method detected and identified more fish diet components than gastric dissection. The data richness of the former technique is accordingly deemed superior to that of the latter (Pompanon et al., 2012). Some postdigestion stomach contents that were difficult to distinguish microscopically could be accurately identified using molecular methods, which require no special skills in anatomy, morphology, or taxonomy. Hu et al. (2014) identified various terrestrial plant species in copepod diets using DNA‐based molecular methods, which could not be identified using traditional morphological methods. Moreover, HTS could produce a sufficient number of sequences for diet analysis even when it over‐represented predator DNA. In this way, it is a very useful technique for obtaining detailed information on small marine organisms that are difficult to characterize from stomach dissection (Pinol, San Andres, Clare, Mir, & Symondson, 2014). The DNA recovered from feces or gut contents roughly reflects the proportion of dietary biomass ingested, even though the number of sequences may be influenced by digestive processes, sampling techniques, and DNA extraction (Deagle et al., 2009; Pompanon et al., 2012). HTS has been used in the dietary analysis of lobster, fur seal, whale, and other marine animals (Deagle et al., 2009; Ford et al., 2016; O'Rorke et al., 2012). In the present study, a greater diversity of prey was more accurately detected and identified by HTS than by microscopic observations. The abundant bryozoans in the stomach contents of *S. argus* juveniles were identified by both molecular evidence and microscopy, thereby indicating that the results of HTS are reliable and can provide relatively quantitative information. Therefore, “traditional” method based on visual observations can provide more direct information and can also be an important means of verification for other new methods, just as HTS in our study (Pompanon et al., 2012).

However, many other prey taxa that were overlooked by microscopic observation were detected through HTS. These included ciliates, polychaetas, and green algae, which were either too small to be detected or too difficult to accurately identify microscopically after digestion. The soft bodied organisms will have a rapidly digestion process with no visible gut remains, so this type of food sources was often neglected by microscopic observation while hard bodied organisms such as copepods with their exoskeleton are often remained identifiable in the gut even after several hours’ ingestion (Scholz, Matthews, & Feller, 1991; Weber & Traunspurger, 2014). As a result, the degeneration of soft bodied prey DNA might be more rapidly than hard bodied prey in digestive process, such as nematodes, which was numerically dominated in benthic meiofaunal community and often was ingested by fish (Schuckel et al., 2013; Weber & Traunspurger, 2015) were rarely founded in our results by HTS detection. While considering the presence of algae and free‐living ciliates in our results, which are typically smaller than nematodes, we assumed that the fish might have the anatomical features to eat soft‐bodied animals. Both methods, especially for more sensitive HTS, might not recovery the complete food sources of *S. argus* due to the rapidly digestive preys with soft bodies. Nevertheless, HTS provides us a more comprehensive perspective and more details on the food sources of fish than traditional morphological identification. The complex composition of the juvenile *S. argus* diet suggests that these fish are omnivorous. This conclusion is consistent with the findings of most previous studies that have used morphological gut content identification and culture observation for their analyses (Gupta, 2016; Sivan & Radhakrishnan, 2011; Wongchinawit & Paphavasit, 2009). In most cases, where many different food sources are available and abundant in the natural environment, juvenile fish of *S. argus* show a preference for planktonic unicellular algae (Gandhi, 2002). Wongchinawit (2007) reported that there is an ontogenic shift in the diet of *S. argus* from microphytoplankton and protozoa in the larval stage to benthic organisms in the adult stage. In addition, the oral anatomy of the juvenile fish (body length range: 1–2 cm) is best suited for the ingestion of microphytoplankton in the water column, their prey size is limited by the mouth gape, which varies from 0.11 to 0.28 cm, villiform type teeth and short gill rakers also limited their ability to catch and chew larger prey (Wongchinawit & Paphavasit, 2009). Nevertheless, the predominant prey species of the juvenile *S. argus* examined in the present study were bryozoans, ciliates, polychaetae, and green algae, all of which are common benthic or sessile fouling organisms (Beech & Landers, 2002; Marroig & Reis, 2011; Tovar‐Hernandez, Mendez, & Villalobos‐Guerrero, 2009; Watson, Scardino, Zalizniak, & Shimeta, 2015). Therefore, as an adaptation to anthropogenic habitat modification, it is assumed that the juvenile fish have shifted from planktonic to benthic feeding.

*Scatophagus argus* is an omnivorous and opportunistic feeder, and therefore, the variation in its dietary composition is related to the abundance of prey in addition to its oral anatomy (Gandhi, 2002). The fish select prey according to food availability and seek a balance between feeding effort and energy gain (Gupta, 2016). Among the major fouling organisms in Daya Bay (Fang & Yan, 2004), bryozoans such as *Bowerbankia* grow copiously on the surfaces of wharfs and buoys and can be readily consumed by fish and other predators through scraping (Marchini, Cunha, & Occhipinti‐Ambrogi, 2007; Yan & Huang, 1990). Few studies, however, have demonstrated that juvenile fish, particularly *S. argus*, select bryozoans as their main energy source. Juvenile *S. argus* live mainly in the surface layers and are poor swimmers compared to the adult fish. In our study, bryozoans predominated in the stomach contents of the juveniles, and *Bowerbankia* constituted a high proportion of all prey DNA sequences detected. Because they are so abundant, bryozoans might be important supplementary food sources for juvenile fish. In this study, it was determined that the main ciliates ingested by *S. argus* were *Zoothamnium* and *Acineta*. Wongchinawit and Paphavasit (2009) reported that the diet of adult *S. argus* during the low‐salinity period consisted mainly of *Zoothamnium*. Certain *Zoothamnium* species are free‐living and grow attached to aquatic plants or inanimate substrates. Others are symbionts on the surfaces of many different animals (Clamp & Williams, 2006). *Acineta* are also common benthic ciliates in the coastal area of the South China Sea (Tan et al., 2010). Therefore, the *Zoothamnium* and *Acineta* in the stomach contents of juvenile *S. argus* may have been derived from (a) active ingestion of the hosts of these ciliates, such as bryozoans, copepods, and polychaetae, along with the ciliates, and/or (b) incidental intake along with the hosts. The role of occasional feeding in the food webs and energy pathways in marine ecosystems thus merits further investigation. *Branchiomma* and *Ulva* were identified as the two other main food sources for the juvenile fish in this study. *Branchiomma* are annelids that are widely distributed globally, particularly in coastal lagoons, rocky shores, marinas, piers, and harbors (Bastida‐Zavala, Buelna, De Leon‐Gonzalez, Camacho‐Cruz, & Carmona, 2016). Species of *Ulva,* which are chlorophyte green algae, are also common benthic organisms in Daya Bay (Qiu, 2015). Both *Branchiomma* and *Ulva* are readily obtained by predators and herbivores. In this study, with the exception of some diatoms (1.77%) and dinoflagellates (0.53%), few unicellular algae were detected in the dietary composition of *S. argus* juveniles. Changes in dietary composition could therefore be the result of variations in food availability in the wharf. In unfavorable environments, organisms might expand their feeding range and select alternative food sources (Quéméré et al., 2013).

The feeding shifts of *S. argus* juveniles may therefore be a response to environmental changes. The original community at the sampling site was destroyed by wharf construction. Subsequently, bryozoans and other fouling organisms superseded and prevailed there and have become more readily accessible to juvenile *S. argus* than unicellular algae. Although *S. argus* requires more plant than animal protein (Song, Su, Liu, & Zhang, 2012), the juveniles prefer as their main food source whatever prey is most available around the wharf, such as sessile bryozoans and other fouling organisms. Our study indicates that *S. argus* responds to the changes in its environment by adopting a flexible feeding strategy. This behavior increases its survival capacity when challenged by anthropogenic disturbances.

*Scatophagus argus* frequently appears near estuaries, mangrove wetlands, beaches, and harbors (Gupta, 2016). For this reason, future research should focus on the impact that *S. argus* has on the abundance and diversity of its prey in these areas. The dietary shifts of *S. argus* suggest that the predation pressure of juvenile fish has switched from plankton to benthic fouling organisms in an artificial environment. This transformation will affect the energy and matter pathways within this coastal region. With continuing expansions in development and exploration, human disturbances will occur more frequently in marine ecosystems, making this shift in predation more common in the future. Our study indicates that changes in the biodiversity and dietary compositions of native animals are effective ways of assessing the impact of anthropogenic disturbances on marine ecosystems. These indices might serve as signals of ecological alteration. Nevertheless, given that our sampling time was very short (two nights), it remains to be determined whether the benthic feeding habit of *S. argus* juveniles will continue over the long term. More extensive and longer studies are thus warranted to determine the far‐reaching impact of human activity on the feeding behavior of fish in this and other marine ecosystems.

## CONFLICT OF INTEREST

None declared.

## AUTHOR CONTRIBUTIONS

Sheng Liu contributed to the conception of the study; Xianzhi Lin performed the experiments, data analyses, and wrote the manuscript; Sheng Liu, Simin Hu, and Hui Huang contributed significantly to manuscript modification.

## DATA ACCESSIBILITY

The 18S rDNA gene sequences obtained in this study had been deposited at GenBank under accession numbers MH423446**–**MH423480, MH427867**–**MH427873 and MH423481.

## References

[ece34380-bib-0001] Anderson, C. , & Cabana, G. (2009). Anthropogenic alterations of lotic food web structure: Evidence from the use of nitrogen isotopes. Oikos, 118(12), 1929–1939. 10.1111/j.1600-0706.2009.17368.x

[ece34380-bib-0002] Bastida‐Zavala, J. R. , Buelna, A. S. R. , De Leon‐Gonzalez, J. A. , Camacho‐Cruz, K. A. , & Carmona, I. (2016). New records of sabellids and serpulids (Polychaeta: Sabellidae, Serpulidae) from the Tropical Eastern Pacific. Zootaxa, 4184(3), 401–457. https://doi.org/10.11646/zootaxa.4184.3.1 10.11646/zootaxa.4184.3.1 27988772

[ece34380-bib-0003] Beech, C. D. , & Landers, S. C. (2002). Ciliated protozoan colonization of substrates from Dauphin Island, Alabama. European Journal of Protistology, 38(1), 83–89. 10.1078/0932-4739-00840

[ece34380-bib-0004] Blankenship, L. E. , & Yayanos, A. A. (2005). Universal primers and PCR of gut contents to study marine invertebrate diets. Molecular Ecology, 14(3), 891–899. 10.1111/j.1365-294X.2005.02448.x 15723681

[ece34380-bib-0005] Clamp, J. C. , & Williams, D. (2006). A molecular phylogenetic investigation of *Zoothamnium* (Ciliophora, Peritrichia, Sessilida). Journal of Eukaryotic Microbiology, 53(6), 494–498. 10.1111/j.1550-7408.2006.00132.x 17123413

[ece34380-bib-0006] Deagle, B. E. , Kirkwood, R. , & Jarman, S. N. (2009). Analysis of Australian fur seal diet by pyrosequencing prey DNA in faeces. Molecular Ecology, 18(9), 2022–2038. 10.1111/j.1365-294X.2009.04158.x 19317847

[ece34380-bib-0007] Fang, F. , & Yan, T. (2004). Status quo and prospects of marine fouling studies in south China sea. Journal of Tropical Oceanography (In Chinese with English abstract), 23(1), 76–85.

[ece34380-bib-0008] Ford, M. J. , Hempelmann, J. , Hanson, M. B. , Ayres, K. L. , Baird, R. W. , Emmons, C. K. , … Park, L. K. (2016). Estimation of a killer whale (*Orcinus orca*) population's diet using sequencing analysis of DNA from feces. PLoS ONE, 11(1), e0144956 10.1371/journal.pone.0144956 26735849 PMC4703337

[ece34380-bib-0009] Gandhi, V. (2002). Studies on the food and feeding habits of cultivable butterfish *Scatophagus argus* (Cuv. and Val.). Journal of the Marine Biological Association of India, 44(1–2), 115–121.

[ece34380-bib-0010] Gupta, S. (2016). An overview on morphology, biology, and culture of Spotted scat *Scatophagus argus* (Linnaeus, 1766). Reviews in Fisheries Science and Aquaculture, 24(2), 203–212. 10.1080/23308249.2015.1119800

[ece34380-bib-0011] Hu, S. M. , Guo, Z. L. , Li, T. , Carpenter, E. J. , Liu, S. , & Lin, S. J. (2014). Detecting in situ copepod diet diversity using molecular technique: Development of a copepod/symbiotic ciliate‐excluding eukaryote‐inclusive PCR protocol. PLoS ONE, 9(7), e103528 10.1371/journal.pone.0103528 25058323 PMC4110036

[ece34380-bib-0012] Huang, X. P. , Zhang, J. P. , & Jiang, Z. J. (2015). Eco‐environmental effects of nutrients input caused by human activities on the semi‐enclosed bay and its management strategy. Advances in Earth Science (In Chinese with English abstract), 30(9), 961–969.

[ece34380-bib-0013] King, R. A. , Read, D. S. , Traugott, M. , & Symondson, W. O. C. (2008). Molecular analysis of predation: A review of best practice for DNA‐based approaches. Molecular Ecology, 17(4), 947–963. 10.1111/j.1365-294X.2007.03613.x 18208490

[ece34380-bib-0014] Leray, M. , Meyer, C. P. , & Mills, S. C. (2015). Metabarcoding dietary analysis of coral dwelling predatory fish demonstrates the minor contribution of coral mutualists to their highly partitioned, generalist diet. PeerJ, 3, e1047 10.7717/peerj.1047 26137428 PMC4485734

[ece34380-bib-0015] Maloy, A. P. , Culloty, S. C. , & Slater, J. W. (2013). Dietary analysis of small planktonic consumers: A case study with marine bivalve larvae. Journal of Plankton Research, 35(4), 866–876. 10.1093/plankt/fbt027

[ece34380-bib-0016] Marchini, A. , Cunha, M. R. , & Occhipinti‐Ambrogi, A. (2007). First observations on bryozoans and entoprocts in the Ria de Aveiro (NW Portugal) including the first record of the Pacific invasive cheilostome *Tricellaria inopinata* . Marine Ecology‐an Evolutionary Perspective, 28(s1), 154–160. 10.1111/j.1439-0485.2007.00173.x

[ece34380-bib-0017] Marroig, R. G. , & Reis, R. P. (2011). Does biofouling influence *Kappaphycus alvarezii* (Doty) Doty ex Silva farming production in Brazil? Journal of Applied Phycology, 23(5), 925–931. 10.1007/s10811-010-9602-y

[ece34380-bib-0018] Moreno‐Valcarcel, R. , Oliva‐Paterna, F. J. , Bevilacqua, S. , Terlizzi, A. , & Fernandez‐Delgado, C. (2016). Long‐term effects of tidal restriction on fish assemblages in east Atlantic coastal marshlands. Marine Ecology Progress Series, 543, 209–222. 10.3354/meps11578

[ece34380-bib-0019] O'Rorke, R. , Lavery, S. , & Jeffs, A. (2012). PCR enrichment techniques to identify the diet of predators. Molecular Ecology Resources, 12(1), 5–17. 10.1111/j.1755-0998.2011.03091.x 22145916

[ece34380-bib-0020] Paradis, V. , Sirois, P. , Castonguay, M. , & Plourde, S. (2012). Spatial variability in zooplankton and feeding of larval Atlantic mackerel (*Scomber scombrus*) in the southern Gulf of St. Lawrence. Journal of Plankton Research, 34(12), 1064–1077. 10.1093/plankt/fbs063

[ece34380-bib-0021] Pinol, J. , San Andres, V. , Clare, E. L. , Mir, G. , & Symondson, W. O. C. (2014). A pragmatic approach to the analysis of diets of generalist predators: The use of next‐generation sequencing with no blocking probes. Molecular Ecology Resources, 14(1), 18–26. 10.1111/1755-0998.12156 23957910

[ece34380-bib-0022] Pompanon, F. , Deagle, B. E. , Symondson, W. O. C. , Brown, D. S. , Jarman, S. N. , & Taberlet, P. (2012). Who is eating what: Diet assessment using next generation sequencing. Molecular Ecology, 21(8), 1931–1950. 10.1111/j.1365-294X.2011.05403.x 22171763

[ece34380-bib-0023] Prudente, B. D. , Carneiro‐Marinho, P. , Valente, R. D. , & Montag, L. F. D. (2016). Feeding ecology of *Serrasalmus gouldingi* (Characiformes: Serrasalmidae) in the lower Anapu River region, Eastern Amazon, Brazil. Acta Amazonica, 46(3), 259–269. 10.1590/1809-4392201600123

[ece34380-bib-0024] Qiu, Y. W. (2015). Bioaccumulation of heavy metals both in wild and mariculture food chains in Daya Bay, South China. Estuarine Coastal and Shelf Science 163, 7–14. 10.1016/j.ecss.2015.05.036

[ece34380-bib-0025] Quéméré, E. , Hibert, F. , Miquel, C. , Lhuillier, E. , Rasolondraibe, E. , Champeau, J. , … Chikhi, L. (2013). A DNA metabarcoding study of a primate dietary diversity and plasticity across its entire fragmented range. PLoS ONE, 8(3), e58971. 23527060 10.1371/journal.pone.0058971 PMC3602585

[ece34380-bib-0026] Riemann, L. , Alfredsson, H. , Hansen, M. M. , Als, T. D. , Nielsen, T. G. , Munk, P. , … Castonguay, M. (2010). Qualitative assessment of the diet of European eel larvae in the Sargasso Sea resolved by DNA barcoding. Biology Letters, 6(6), 819–822. 10.1098/rsbl.2010.0411 20573615 PMC3001378

[ece34380-bib-0027] Robert, D. , Levesque, K. , Gagne, J. A. , & Fortier, L. (2011). Change in prey selectivity during the larval life of Atlantic cod in the southern Gulf of St Lawrence. Journal of Plankton Research, 33(1), 195–200. 10.1093/plankt/fbq095

[ece34380-bib-0028] Scholz, D. S. , Matthews, L. L. , & Feller, R. J. (1991). Detecting selective digestion of meiobenthic prey by juvenile spot *Leiostomus xanthurus* (pisces) using immunoassays. Marine Ecology Progress Series, 72(1–2), 59–67. 10.3354/meps072059

[ece34380-bib-0029] Schuckel, S. , Sell, A. F. , Kihara, T. C. , Koeppen, A. , Kroncke, I. , & Reiss, H. (2013). Meiofauna as food source for small‐sized demersal fish in the southern North Sea. Helgoland Marine Research, 67, 203–218.

[ece34380-bib-0030] Sivan, G. , & Radhakrishnan, C. K. (2011). Food, feeding habits and biochemical composition of *Scatophagus argus* . Turkish Journal of Fisheries and Aquatic Sciences, 11(4), 603–608.

[ece34380-bib-0031] Song, Y. , Su, M. L. , Liu, N. X. , & Zhang, J. B. (2012). Studies on low temperature resistance and nutritional needs of *Scatophagus argus* juveniles. Journal of Shanghai Ocean University (In Chinese with English abstract), 21(5), 715–719.

[ece34380-bib-0032] Stoeck, T. , Bass, D. , Nebel, M. , Christen, R. , Jones, M. D. M. , Breiner, H. W. , & Richards, T. A. (2010). Multiple marker parallel tag environmental DNA sequencing reveals a highly complex eukaryotic community in marine anoxic water. Molecular Ecology, 19(Suppl. 1), 21–31. 10.1111/j.1365-294X.2009.04480.x 20331767

[ece34380-bib-0033] Tan, Y. , Huang, L. , Huang, X. , Su, Q. , Shi, X. , & Huang, J. (2010). The relationships between ciliate composition, abundance and environmental factors in Sanya gay coral reef waters. Acta Ecologica Sinica (In Chinese with English abstract), 30(24), 6835–6844.

[ece34380-bib-0034] Tovar‐Hernandez, M. A. , Mendez, N. , & Villalobos‐Guerrero, T. F. (2009). Fouling polychaete worms from the southern Gulf of California: Sabellidae and Serpulidae. Systematics and Biodiversity, 7(3), 319–336. 10.1017/S1477200009990041

[ece34380-bib-0035] Watson, M. G. , Scardino, A. J. , Zalizniak, L. , & Shimeta, J. (2015). Colonisation and succession of marine biofilm‐dwelling ciliates in response to environmental variation. Aquatic Microbial Ecology, 74(2), 95–105. 10.3354/ame01731

[ece34380-bib-0036] Weber, S. , & Traunspurger, W. (2014). Consumption and prey size selection of the nematode Caenorhabditis elegans by different juvenile stages of freshwater fish. Nematology, 16, 631–641. 10.1163/15685411-00002793

[ece34380-bib-0037] Weber, S. , & Traunspurger, W. (2015). The effects of predation by juvenile fish on the meiobenthic community structure in a natural pond. Freshwater Biology, 60(11), 2392–2409. 10.1111/fwb.12665

[ece34380-bib-0038] Wongchinawit, S. (2007). Feeding ecology of spotted scat Scatophagus argus, Linnaeus in mangrove forests, Pak Phanang Estuary, Nakhon Si Thammarat Province (PhD thesis). Chulalongkorn University, Thailand *The Natural History Journal of Chulalongkorn University*, 9(2), 143–169. 10.1128/AEM.68.2.989-994.2002

[ece34380-bib-0039] Wongchinawit, S. , & Paphavasit, N. (2009). Ontogenetic niche shift in the Spotted Scat, *Scatophagus argus*, in Pak Phanang Estuary, Nakhon Si Thammarat Province, Thailand. The Natural History Journal of Chulalongkorn University, 9(2), 143–169.

[ece34380-bib-0040] Yan, S. K. , & Huang, Z. G. (1990). Study of fouling organisms in Daya Bay, China. Biofouling (In Chinese), 2(3), 229–237. 10.1080/08927019009378147

[ece34380-bib-0041] Zhang, H. , & Lin, S. J. (2002). Detection and quantification of *Pfiesteria piscicida* by using the mitochondrial cytochrome b gene. Applied and Environmental Microbiology, 68(2), 989–994. 11823251 10.1128/AEM.68.2.989-994.2002 PMC126730

