# Supplementary material for: Unexpected prey of juvenile spotted scat (Scatophagus argus) near a wharf: The prevalence of fouling organisms in stomach contents
Source: Ecol Evol. 2018 Jul 30;8(16):8547–54. doi: 10.1002/ece3.4380 (PMC6145014; doi:10.1002/ece3.4380)
Supplement: Supplementary file 1 [file ECE3-8-8547-s001.docx]

**Tab 1** Taxonomic distribution and closest relative species of OTUs retrieved from stomach contents of *S. argus* juveniles

| ID | Total sequences recovered | Highest taxon | Lowest consensus taxon | Lowest classification level | Similarity | Closest relative | Accession number |
| --- | --- | --- | --- | --- | --- | --- | --- |
| H-1 | 2471 | Annelida | *Branchiomma* | Genus | 99% | *Branchiomma* sp. | EF116212.1 |
| H-2 | 10 | Annelida | *Parasabella saxicola* | Species | 100% | *Parasabella saxicola* | KT900323.1 |
| H-3 | 77 | Tunicata | *Styela* | Genus | 98% | *Styela gibbsii* | FM897319.1 |
| H-4 | 517 | Chlorophyta | *Ulva* | Genus | 99% | *Ulva* sp. | KT943572.1 |
| H-5 | 122 | Ciliophora | *Pseudovorticella* | Genus | 98% | *Pseudovorticella paracratera* | DQ662847.1 |
| H-6 | 107 | Ciliophora | *Pseudovorticella* | Genus | 99% | *Pseudovorticella punctata* | DQ190466.1 |
| H-7 | 64 | Ciliophora | *Vaginicola* | Genus | 98% | *Vaginicola* sp. | KJ649621.2 |
| H-8 | 284 | Ciliophora | Vaginicolidae | Family | 96% | *Vaginicola* sp. | KJ649621.2 |
| H-9 | 79 | Ciliophora | *Vaginicola crystallina* | Species | 99% | *Vaginicola crystallina* | AF401521.1 |
| H-10 | 2217 | Ciliophora | *Zoothamnium* | Genus | 100% | *Zoothamnium* sp, | KU363270.1 |
| H-11 | 353 | Ciliophora | *Zoothamnium* | Genus | 99% | *Zoothamnium parahentscheli* | KM887956.1 |
| H-12 | 530 | Ciliophora | *Zoothamnium arcuatum* | Species | 99% | *Zoothamnium arcuatum* | KM887955.1 |
| H-13 | 237 | Ciliophora | Zoothamniidae | Family | 97% | *Zoothamnium* sp. | KU363270.1 |
| H-14 | 200 | Ciliophora | *Zoothamnium* | Genus | 99% | *Zoothamnium grossi* | KM887954.1 |
| H-15 | 77 | Ciliophora | *Hemiophrys* | Genus | 98% | *Hemiophrys macrostoma* | AY102173.1 |
| H-16 | 13 | Ciliophora | Dysteriidae | Family | 96% | *Dysteriidae* sp. | KJ569308.1 |
| H-17 | 15 | Ciliophora | *Ephelota* | Genus | 99% | *Ephelota* sp. | AF326357.1 |
| H-18 | 19 | Ciliophora | Ephelotidae | Family | 97% | *Ephelota* sp. | AF326357.1 |
| H-19 | 3561 | Ciliophora | *Acineta* | Genus | 98% | *Acineta compressa* | FJ865205.1 |
| H-20 | 39 | Dinophyceae | *Protoperidinium* | Genus | 99% | *Protoperidinium tricingulatum* | AB716918.1 |
| H-21 | 27 | Dinophyceae | *Peridinium quinquecorne* | Species | 99% | *Peridinium quinquecorne* | AB246744.1 |
| H-22 | 24 | Dinophyceae | *Prorocentrum* | Genus | 99% | *Prorocentrum koreanum* | KP711353.1 |
| H-23 | 22 | Phaeophyta | *Ectocarpus* | Genus | 99% | *Ectocarpus fasciculatus* | KU752533.1 |
| H-24 | 25 | Bacillariophyta | *Chaetoceros* | Genus | 100% | *Chaetoceros dayaensis* | KM401854.1 |
| H-25 | 30 | Bacillariophyta | *Minutocellus polymorphus* | Species | 100% | *Minutocellus polymorphus* | KF925333.1 |
| H-26 | 45 | Bacillariophyta | Skeletonemataceae | Family | 96% | *Skeletonema menzellii* | AB948147.1 |
| H-27 | 83 | Bacillariophyta | *Cyclotella* | Genus | 99% | *Cyclotella atomus* | DQ514858.1 |
| H-28 | 18 | Bacillariophyta | *Navicula pulchripora* | Species | 99% | *Navicula pulchripora* | KF177774.1 |
| H-29 | 12 | Bacillariophyta | *Cylindrotheca* | Genus | 100% | *Cylindrotheca* sp. | FR865492.1 |
| H-30 | 18 | Bacillariophyta | *Melosira dubia* | Species | 100% | *Melosira dubia* | AB430588.1 |
| H-31 | 51 | Bacillariophyta | *Licmophora* | Genus | 98% | *Licmophora grandis* | EF423411.1 |
| H-32 | 19 | Bacillariophyta | Cyclophorales | Order | 96% | *Cyclophora tenuis* | JN975241.1 |
| H-33 | 42 | Arthropoda | *Harpacticus* | Genus | 99% | *Harpacticus* sp. | EU380285.1 |
| H-34 | 157 | Arthropoda | Tegastidae | Family | 95% | *Tegastes* sp. | EU380286.1 |
| H-35 | 5405 | Bryozoa | *Bowerbankia* | Genus | 99% | *Bowerbankia* sp. | KM373516.1 |
| H-36 | 3791 | Vertebrata | Osteichthyes | Class | 100% | *Ambassis marianus* | KJ774636.1 |
| H-37 | 18 | Labyrinthulomycetes | *Aplanochytrium* | Genus | 100% | *Aplanochytrium* sp. | AB973543.1 |
| H-38 | 17 | Labyrinthulomycetes | NA | NA | 93% | Unknown | AF265331.1 |
| H-39 | 97 | Ciliophora | NA | NA | 91% | Unknown | LK934651.1 |
| H-40 | 45 | Ciliophora | NA | NA | 86% | Unknown | JN867017.1 |
| H-41 | 20 | Arthropoda | NA | NA | 87% | Unknown | EU380305.1 |
| H-42 | 16 | Ciliophora | NA | NA | 87% | Unknown | KC753482.1 |
